# Supplementary material for: Turnkey photonic flywheel in a microresonator-filtered laser
Source: Nat Commun. 2024 Jan 2;15:55. doi: 10.1038/s41467-023-44314-8 (PMC10761980; doi:10.1038/s41467-023-44314-8)
Supplement: Supplementary file 1 — Supplementary Information [file 41467_2023_44314_MOESM1_ESM.pdf]

Supplementary Information for "Turnkey photonic flywheel in a microresonator-filtered laser"

Mingming Nie[1*], Jonathan Musgrave[1], Kunpeng Jia[2,*], Jan Bartos[1], Shining Zhu[2], Zhenda Xie[2,*] and Shu-Wei Huang[1,*]

[1]*Department of Electrical, Computer and Energy Engineering, University of Colorado Boulder, Boulder, Colorado 80309, USA*

[2]*National Laboratory of Solid State Microstructures, School of Electronic Science and Engineering, College of Engineering and Applied Sciences, School of Physics, and Collaborative Innovation Center of Advanced Microstructures, Nanjing University, Nanjing 210093, China*

[*]*Corresponding author: mingming.nie@colorado.edu, jiakunpeng@nju.edu.cn, xiezhenda@nju.edu.cn, shuwei.huang@colorado.edu*

This Supplementary Information for "Turnkey photonic flywheel in a microresonator-filtered laser" provides additional information for the main text.

- In Section I, we compare the dynamics between SBL soliton and conventional soliton when pump frequency is scanned from blue side to red side.
- In Section II, we study the effect of microresonator temperature on the SBL soliton generation including the deterministic selection of soliton number.
- In Section III, we conduct comprehensive theoretical and numerical analysis including the numerical reproduction of turnkey soliton generation.
- In Section IV, we provide the detailed analysis on the perfect SBL soliton crystal generation.
- In Section V, we show the soliton self-healing behavior in the experiment.
- In Section VI, we provide the integrated soliton timing jitter in the experiment.
- In Section VII, we recall the theory for SBL soliton noise suppression.
- In Section VIII, we provide the detailed analysis on the limit of laser and comb frequency noise.
- In Section IX, we provide the detailed analysis on the phase noise of the generated microwave signal, including the SBS frequency shift and comb repetition rate.
- In Section X, we compare the noise performance with the reported soliton microcombs in terms of the fundamental comb linewidth and phase noise of the comb repetition rate.
- In Section XI, we provide the long-term stability of pump detuning and pump frequency shift in the experiment.
- In Section XII, we provide the details of how the PDH error signal is obtained.

I. Different dynamics in the SBL soliton and conventional soliton generation

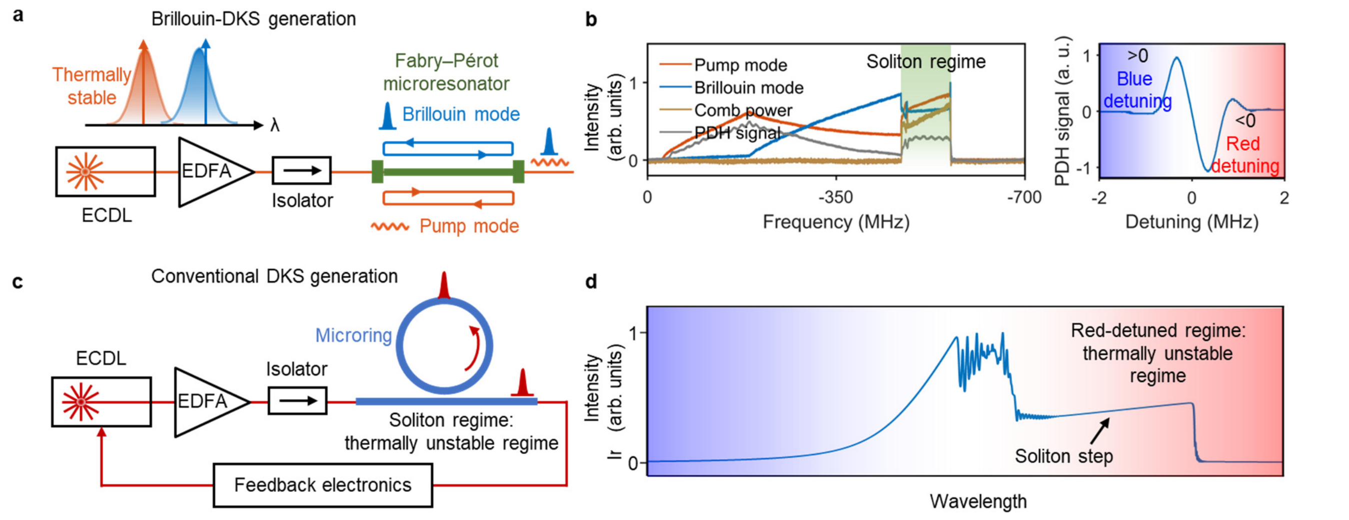

Fig. S1. Different types of DKS generation and sweeping dynamics when pumping the microresonator with a tunable external cavity diode laser (ECDL, single-frequency laser). (a)(b) Brillouin-DKS generation; (c)(d) Conventional DKS generation. (a) Schematic of Brillouin-DKS generation utilizing two-step pumping scheme. (b) Left: experimental pump PDH signal and power evolution of pump mode, Brillouin mode and combs as the ECDL frequency sweeps across the pump resonance from blue to red side. Right: frequency-calibrated pump PDH signal at low pump power. The modulation frequency of the phase modulator is 1 MHz, close to the linewidth of the high-Q microresonator. PDH signal>0: blue detuning, PDH signal<0: red detuning, PDH signal=0, zero detuning (resonance peak). During the sweeping process, stimulated Brillouin laser and Brillouin-DKS are excited. In the two-step pumping scheme, the red-detuned and comb-generating SBL serves as the direct soliton energy source, while the blue-detuned pump compensates the thermal nonlinearity and stabilizes the Brillouin-DKS without sophisticated feedback electronics. When the soliton is generated, the transmitted pump laser power increases. (c) Schematic of conventional DKS generation. (d) Simulated transmission spectrum when the pump laser is scanned from blue side to red side at a proper scanning speed depending on the thermal response. Solitons can form in the red-detuned regime seeded from modulation instability. However, complex electronics are required to stabilize the soliton since pump laser is thermally unstable in the red-detuned regime and will be kicked out immediately from the microresonator due to the perturbation [1].

II. Effect of microresonator temperature on the SBL soliton generation

Figure S2 shows how the microresonator is temperature controlled. Our MMF FP microresonator is mounted in a ceramic sleeve and the sleeve is clamped in the metal mount. Only half of the mount is temperature controlled through a thermoelectric cooler. During the long-term operation, the microresonator temperature can slow drifts due to the lab temperature change.

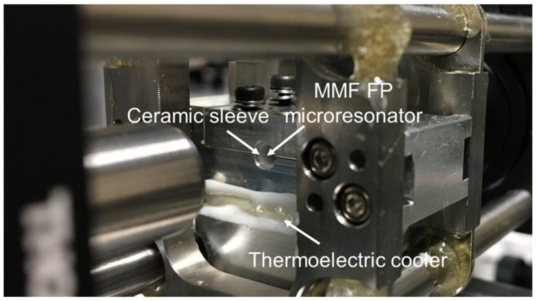

Fig. S2. Picture showing how the microresonator is temperature controlled. The encapsuled MMF FP microresonator in a ceramic sleeve is enclosed in a holder, which is temperature controller by a thermoelectric cooler.

The temperature of our MMF FP microresonator can affect the SBS frequency shift [2,3] and thus the SBL power and SBL detuning. To generate Brillouin-Kerr soliton, the SBS frequency shift should be conditioned slightly larger than the offset frequency between the pump and SBL such that the blue-detuned pump can compensate the thermal nonlinearity of the red-detuned comb generating SBL. In the experiment, the temperature range for soliton generation is around 0.8 K. Figure S3 shows the effect of microresonator temperature on the soliton existing regime by sweeping the ECDL from blue side to red side with a fixed pump power. With slightly higher temperature by ~0.5 K (than the upper boundary temperature for DKS), the SBS frequency shift becomes larger and the maximum Brillouin gain reduces due to the smaller overlapping between the Brillouin gain spectrum and the Brillouin mode resonance. Therefore, the SBL power is not enough for soliton generation before pump drops out from the microresonator (Fig. S3a). With slightly lower temperature (than the lower boundary temperature for DKS) by ~0.1 K and ~0.5 K, the SBS frequency shift becomes smaller. Figures S3b and S3c show the example when the SBS frequency shift is smaller than the offset frequency between the pump and SBL. The maximum SBL power can be achieved with small blue pump detuning. After that the SBL quenches since it is below the threshold and the pump continues sweeping towards red side. Without energy conversion to SBL, the intra-microresonator pump power is large enough to excite the pump chaotic combs from the modulation instability before dropping out from the microresonator. When the microresonator temperature deviates too much from the range for DKS generation, the SBS frequency shift is much larger or smaller than the offset frequency between the pump and SBL. Therefore, SBL can not be generated during the pump sweeping process due to the small Brillouin gain, as shown in Fig. S3d.

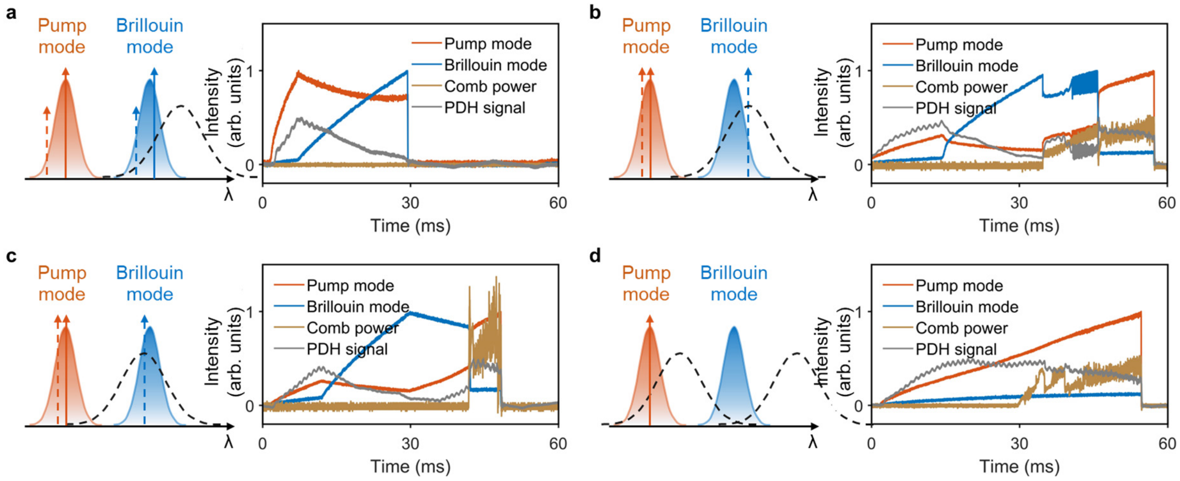

Fig. S3. Effect of microresonator temperature on the soliton existing regime. (a) With higher temperature by ~0.5 K. (b) With lower temperature by ~0.1 K. (c) With lower temperature by ~0.5 K. The SBL power might be enough, but the SBL detuning is not correct at near blue side. (d) With lower temperature or higher temperature by ~2 K. For each subfigure, left: the relationship between the SBS frequency shift and the pump-Brillouin mode offset frequency. The dashed black lines represent the SBS gain spectrum. The orange solid lines represent the pump laser before dropping out from the microresonator. The dashed orange and blue lines represent the pump and generated Brillouin laser in the sweeping process. Right: real-time evolution of pump mode power, Brillouin mode power, comb power and PDH signal.

Figure S4 shows the soliton number dynamics when changing the microresonator temperature in the soliton existing regime. The ECDL is swept from blue side to red side. By changing the microresonator temperature, the SBS frequency shift is changed thus both the SBL detuning and thermal equilibrium condition are changed when pump detuning is near zero. With two intracavity lasers compensated thermal effect, the SBL soliton existing regime is no longer degenerate due to the XPM effect [4–7]. The soliton steps showing the soliton number switching are clearly observed in Fig. S4. By changing the temperature, we can deterministically select the soliton number from 3 to 1 before pump power drops or pump laser exits the microresonator at what time the pump detuning is around zero detuning. Therefore, in our microresonator-filtered fiber laser, we can deterministically select the soliton number by changing the microresonator temperature.

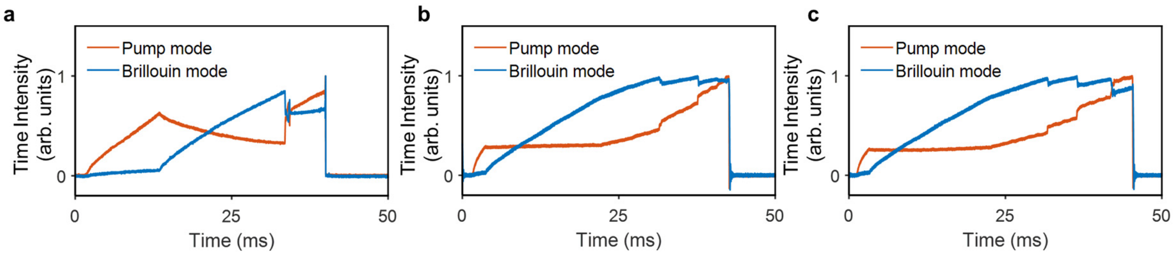

Fig. S4. Soliton dynamics by sweeping the pump laser frequency under different microresonator temperatures. (a) the final soliton number (before pump power drops or pump laser exits the microresonator) is 3; (b) the final soliton number is 2; (c) the final soliton number is 1.

III. Theoretical and numerical analysis

First, we model the formation and dynamics of the Brillouin-Kerr frequency comb for the *externally* pumped case to verify the importance of the Brillouin frequency shift in the two-step pumping scheme. We employ a set of coupled mode equations in frequency domain which combines both the three-wave interaction for the SBS process and four-wave-mixing in both pump and Brillouin mode families during the comb-generating process. We also consider the thermal effect in our FFP microresonator, which causes the thermo-optic drift of the mode resonance. Therefore, the theoretical model of Brillouin-Kerr resonator takes the form:

$$\frac{\delta \tilde{P}_\mu}{\delta t} = \left(-\frac{\gamma_P}{2} + i\sigma_p - i\beta_p\Delta T + i\left[D_{p,\mu}\right]\right)\tilde{P}_n - ig_b\delta_0 bB_0 - ig_{pK_1}\mathcal{F}^{-1}\{|P|^2P\}_\mu - i2g_{K_2}\mathcal{F}^{-1}\{|B|^2P\}_\mu + \delta_0\sqrt{\kappa_P}s_{in}, \quad \text{(S1)}$$

$$\frac{\delta \tilde{B}_\mu}{\delta t} = \left(-\frac{\gamma_B}{2} + i\sigma_B - i\beta_B\Delta T + i\left[D_{B,\mu}\right]\right)\tilde{B}_n - ig_b\delta_0 P_0 b^* - ig_{BK_1}\mathcal{F}^{-1}\{|B|^2B\}_\mu - i2g_{K_2}\mathcal{F}^{-1}\{|P|^2B\}_\mu, \quad \text{(S2)}$$

$$\frac{\delta b}{\delta t} = \left(-\frac{\Gamma_b}{2} + i\sigma_b + i\Delta\Omega\right)b - ig_b\delta_0 P_0 B_0^*, \quad \text{(S3)}$$

$$\frac{\Delta T}{\delta t} = -\frac{1}{\tau_T}\Delta T + c_{T,p}\sum_\mu\left|\tilde{P}_\mu\right|^2 + c_{T,B}\sum_\mu\left|\tilde{B}_\mu\right|^2, \quad \text{(S4)}$$

where $\tilde{P}_\mu$, $\tilde{B}_\mu$, b are the fields of pump mode, Brillouin mode and acoustic wave, respectively (μ is the mode number). $\gamma_{p(B)}$ is the photon decay rate for pump (Brillouin) modes including both intrinsic and external losses. Γ_b is the phonon decay rate. g_b is the is the coupling coefficient between the optical and acoustic modes in the SBS process, while $g_{p(B)K_1}$ and g_{K_2} are the self and cross phase modulation coefficients respectively. The dispersion $D_{p(B),\mu}$ relates the photon mode frequency, $\omega_{\mu,p(B)}$, to the center frequency, $\omega_{0,P(B)}$, by the relation $\omega_{\mu,P(B)} = \sigma_{P(B)} + \omega_{0,p(B)} + D_{p(B),\mu}$. The coupled equations are with reference to the detuning values $\sigma_{p(B)} = \omega_{p,B} - \omega_{0,p(B)}$ and $\sigma_b = \Omega_b - \Omega_0$ subject to the phase matching relationship $\sigma_p - (\sigma_B + \sigma_b) = 0$. The term $\Delta\Omega$ represents the frequency offset between the SBS wave and the central Brillouin mode frequency. To facilitate numerical simulation the inverse Fourier, transform operator, $\mathcal{F}^{-1}$, is used to calculate the four wave mixing processes between pump and Brillouin modes. κ_P and s_{in} correspond to the coupling rate and power of the external pump. The fourth equation describes the intracavity power induced temperature change ΔT from the power absorption. In the equation, $c_{T,p(B)}$ and τ_T are the modal thermal absorption coefficient and thermal relaxation time respectively. The temperature change will cause the frequency drift of the pump and Brillouin mode due to the refractive index change through thermo-optic effect. The frequency shift can be expressed as $\Delta\omega_{T,p(B)} = \beta_{p(B)}\Delta T$, where $\beta_{P(B)}$ is the thermo-optic coefficient.

Using the above set of equations, we numerically calculate 2048 modes of both the Brillouin and pump mode families for the *externally* driven thermally stable Brillouin Kerr-Soliton generation, as shown in Fig. S5. The parameters used for simulation are: $\kappa_p = \kappa_B = 2\pi * 0.4$ MHz, $\gamma_{0,p} = \gamma_{0,B} = 2\pi * 0.7$ MHz, $\Gamma = 2\pi * 30$ MHz. The mode frequencies were defined $\Omega = 2\pi * 10.5$ GHz and $\omega_P = 2\pi * 193.73$ THz. The dispersion parameters were set for the FSR as $D_{1,p} = D_{1,B} = 10$ GHz, GVD $D_{2,p} = D_{2,B} = 0.57$ kHz and high-order dispersions are neglected. $c_{T,p} = c_{T,B} = 50\ K/J \cdot GHz$, $\gamma_{abs,p} = \gamma_{abs,B} = 5\,K/J$, $\beta_p = \beta_B = 2\pi * 100\ kHz/K$, and $1/\tau_T = 10$ MHz. $V_{eff,B} = V_{eff,p} = 3.181E^{-12}\mu m^3$.

Figure S5a shows the evolution of stable Brillouin-DKS generation process in the presence of thermo-optic drift when pump frequency is scanned from the blue side to red side. If the SBS frequency shift is set to be slightly larger than the offset frequency ($\Delta\Omega$) between pump and Brillouin center mode resonances, a blue-detuned pump and red-detuned SBL can stably coexist in the microresonator. When the soliton is generated, the intracavity power won't drop as in Fig. S1d but will increase (Fig. S5a) due to the two coexisting lasers, leading to thermal nonlinearity compensation. The numerical results in Fig. S5a

agree with the experimental ones in Fig. S1b, verifying the accuracy of our model. If the SBS frequency shift is set to be smaller than the offset frequency (ΔΩ) between pump and Brillouin center mode resonances, the SBL is blue-detuned, which leads to chaotic comb generation or continuous-wave generation, as shown in Fig. S5b and S5c. Of note, in the experiment the relationship between the SBS frequency shift and the offset frequency (ΔΩ) is adjusted by the microresonator temperature which mainly changes the SBS frequency shift. However, in the simulation, the relationship is adjusted only by changing the offset frequency (ΔΩ) in Eq. (S3).

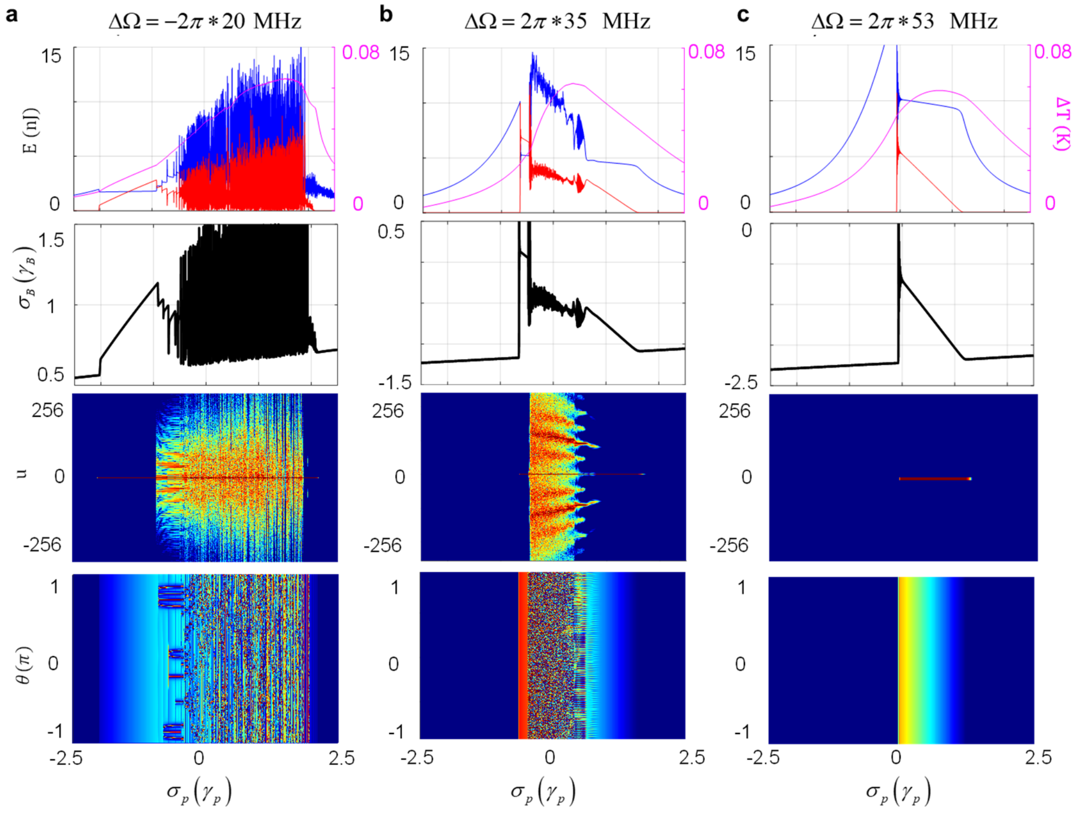

Fig. S5. Dynamics of externally pumped Brillouin-Kerr microresonator when pump is scanned from blue side to red side. The pump detuning is normalized by the cavity linewidth of pump mode. (a) Soliton generation; (b) chaotic comb generation; (c) continuous-wave generation. The first row shows the evolution of intracavity energy (for both center modes, red: SBL, blue: pump) and the intracavity temperature (magenta). The second row shows the evolution of SBL detuning (normalized by the cavity linewidth of Brillouin mode). The third and fourth rows show the spectral and temporal evolution of Brillouin mode families, respectively.

Next, we model the microresonator-filtered laser to numerically verify the formation of soliton attractor. To form a laser cavity, the input and output of the microresonator are connected through the active gain and a narrowband spectral filter. Since only the pump center mode (single frequency) $\tilde{P}_0$ experiences laser gain, the saturable gain g reads as

$$g = \frac{g_0}{1+\frac{\kappa_P|\tilde{P}_0|^2}{P_{sat}}} \qquad \text{(S5)}$$

where g_0 represents the small signal gain of the fiber amplifier and P_{sat} is the saturation power of the EDFA. Additionally, the refractive index change of the EDFA, governed by the excited pump ions, can cause the lasing frequency shift in the large fiber cavity, which can equivalently act as a frequency-sweeping laser as in the above-mentioned sweeping case. Therefore, the shifted pump frequency will lead to the pump detuning change, which is modelled as $\sigma_p = |s_{in}|^2\xi + \sigma_{p,0}$, where ξ is a fitting parameter set to model the external cavity red shift and $\sigma_{p,0}$ is the initial cold cavity detuning of the pump mode. The microresonator-filtered laser cavity model is completed by replacing the external pump term in Eq. S(1) with the amplified power of pump center mode $|s_{in}| = \sqrt{g|P_0|^2}$. In the simulation, we set the small signal gain to $g_0 = 2.55$ 1/m and $P_{sat} = 1\ W$ for a 2-m EDFA.

Numerical demonstration of soliton attractor As shown in Fig. S6a-c with $\beta_p = \beta_B = 2\pi * 100\ kHz/K$, Brillouin-DKS can self-start from seeded quantum noise. The pump is spontaneously attracted to near the resonance peak for maximizing the output power. After some oscillations (power oscillations in Fig. S6a, horizontal oscillations in Fig. S6c and Supplementary Movie S1 online) caused by both the gain effect and thermal effect, the Brillouin-DKS is eventually attracted to the stable magenta star point in the detuning diagram (Fig. S6c), where pump is blue detuned and SBL is red detuned. The numerical result in Fig. S6c matches well the schematic soliton attractor in the main text. Additionally, we numerically simulate the case with a 10 times larger thermo-optic coefficient. As shown in Fig. S6c-e, the larger thermal nonlinearity can be well compensated, and the system can eventually evolve into the stable Brillouin-DKS state (green star point in Fig. S6c). This verifies that our microresonator-filtered laser configuration is general and can be applied to different microresonators.

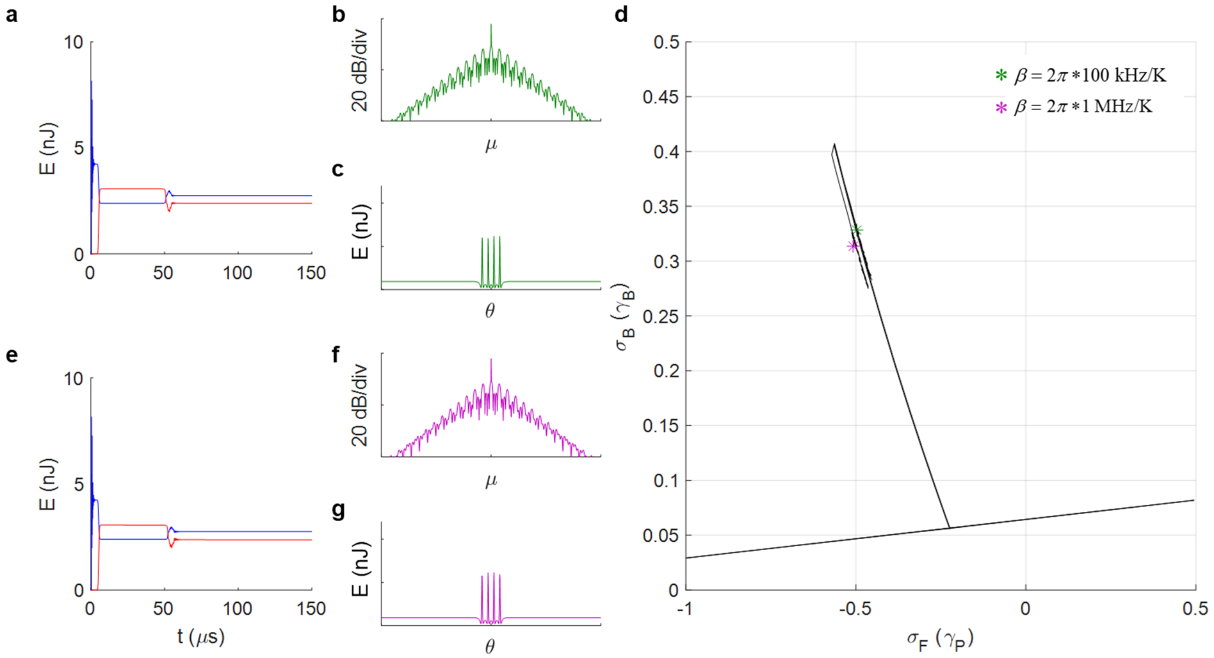

Fig. S6. Numerical demonstration of self-starting soliton in a microresonator-filtered fiber laser. (a)(e) Energy evolution of both pump (blue) and SBL (red), (b)(f) stable soliton spectrum, (c)(g) stable temporal waveform, and (d) the detuning evolution during the self-staring process to a stable soliton state. (a-c) $\beta_p = \beta_B = 2\pi * 100\,kHz/K$; (e-g) $\beta_p = \beta_B = 2\pi * 1\,MHz/K$.

Soliton attractor determined by the SBS frequency shifts Our simulation model reveals that the cavity attractor is mainly determined by the SBS frequency shift, which finally changes the detunings and intracavity powers. Similar to Fig. S5, by adjusting the SBS frequency shift, the final stable cavity states vary from sing-frequency SBL generation to Brillouin-DKS generation as shown in Fig. S7.

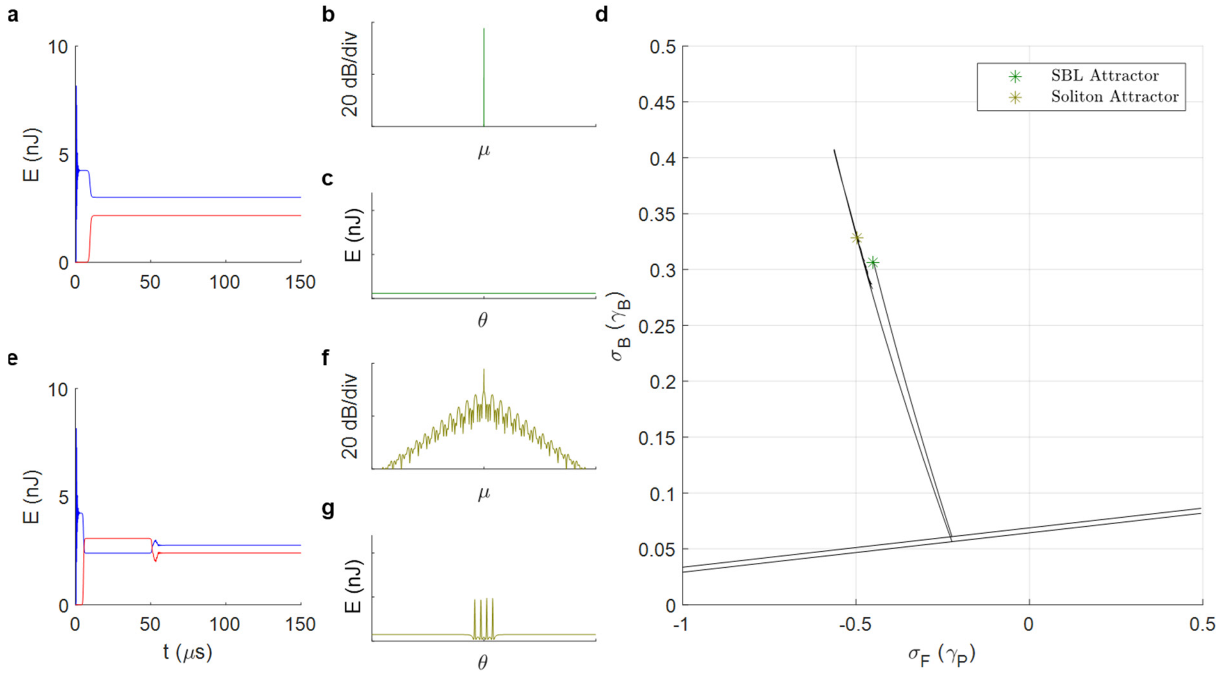

Fig. S7. Cavity attractor under different SBS frequency shifts (Supplementary Movie 2 online). (a)(e) Energy evolution of both pump (blue) and SBL (red), (b)(f) stable soliton spectrum, (c)(g) stable temporal waveform, and (d) the detuning evolution during the self-staring process to a stable soliton state. (a-c) $\Delta\Omega = -2\pi * 30\ MHz$; (e-g) $\Delta\Omega = -2\pi * 20\ MHz$.

Soliton attractor insensitive to the large fiber cavity change Different from the reported laser cavity soliton where the balance of the thermal nonlinearity in the microresonator is realized by the gain nonlinearity, for our Brillouin-DKS in the microresonator-filtered laser, the active gain does not play a role in the thermal nonlinearity compensation. In fact, the thermal nonlinearity is compensated only in the microresonator by the two coexisting pump and Brillouin laser. The large fiber cavity here only provides the equivalent single frequency pump sweeping process resulting from the refractive index increase and thermal expansion. Therefore, it allows self-starting cavity dynamics to be independent of the initial feedback phase or fiber cavity length.

By implementing the two-step pumping scheme in the microresonator, the generated Brillouin-DKS is insensitive to the large fiber cavity change, either the length change or gain change. The cavity length change of the large fiber cavity can lead to the pump detuning change. Thanks to the SBS process, the SBL detuning change can be largely reduced (see Section VII for details) from the pump detuning. Therefore, even strong pump frequency modulation (or strong cavity length modulation) does not adjust the final soliton attractor significantly.

The EDFA gain change will also lead to the pump detuning change as well as the intra-microresonator power change. Thanks to the thermal nonlinearity compensated by the two coexisting lasers, the system can tolerate a large pump power change through thermal self-organization. Therefore, the Brillouin-DKS can exist over a broad range of EDFA gain values. Experimentally, the EDFA pump power tolerance of our deterministic turnkey process is ±100 mW at 1.5 W. In simulation, we see that the integrated gain can change by $\pm$ 0.25 dB while remaining in the same soliton state, which corresponds to a $\pm$6% change in the EDFA pump power agreeing well with the experimentally observed $\pm$6.5%. In all, the Brillouin-DKS has strong perturbative immunity and excellent long-term stability.

IV. Perfect SBL soliton crystal generation

We attribute the turnkey perfect soliton crystal (PSC) generation in our microresonator-filtered laser to the equally spaced potential well [8] created by co-lasing pump mode lasers due to the insufficient filtering effect of the BPF. Figure S7 shows the spectrum of the pump mode at the output of the EDFA when SBL soliton is generated. Besides the main lasing signal, there are three other lasing signals for the pump mode. The co-lasing signals will create equally spaced potential well ranging from 1 FSR to 3 FSRs through XPM effect, attract soliton to be equally spaced in time domain and lead to PSC. Of note, pump mode FSR is almost the same with the Brillouin mode FSR in our FP microresonator made of graded-index fiber.

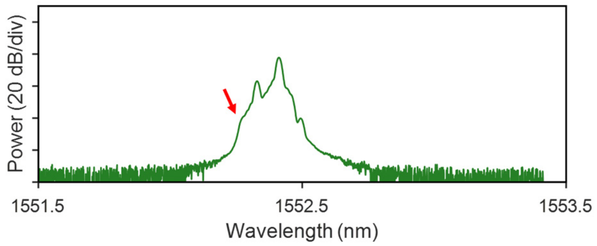

Fig. S8. Pump mode spectrum. One of the pump mode lasers is buried due to the limited resolution of the optical spectrum analyzer (YOKOGAWA, AQ6370D), as indicated by the red arrow. The Brillouin mode suppression is larger than 40 dB so that SBL comb lines can not be observed clearly.

V. Soliton self-healing

Figure S9 shows soliton can self-heal itself from vibration and temperature change.

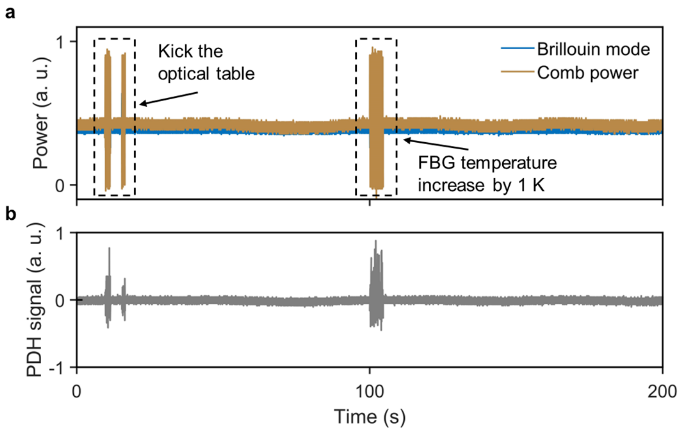

Fig. S9. Soliton self-healing from perturbation. (a) Temporal evolution of comb power and Brillouin mode power. (b) Temporal evolution of PDH signal.

VI. SBL soliton timing jitter

As shown in Fig. S10, the timing jitter integrated from 18 kHz to 1 MHz is 1 fs and timing jitter integrated from 550 Hz to 1 MHz is single optical cycle (~5.2 fs).

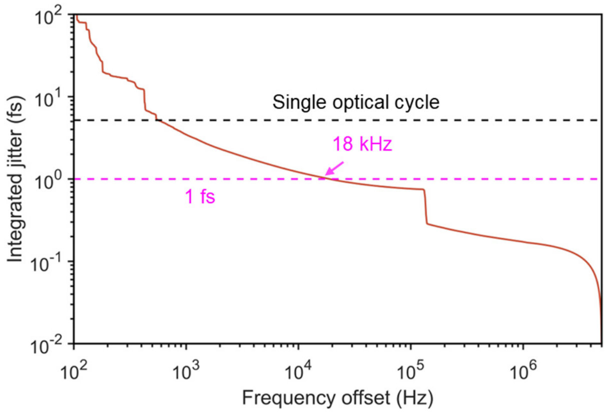

Fig. S10. Integrated timing jitter. The timing jitter integrated from 18 kHz to 1 MHz is 1 fs and timing jitter integrated from 550 Hz to 1 MHz is single optical cycle (~5.2 fs).

VII. Theory for SBL soliton noise suppression

SBL and comb linewidth narrowing When the Brillouin mode resonance peak match the SBS gain peak, the SBL narrowing factor r is determined by $r = (1 + \Gamma_b/\gamma)^2$ [9–11], where Γ_b is the Brillouin gain bandwidth and γ is the Brillouin mode cavity linewidth. Therefore, larger Q factor can lead to better SBL noise suppression and larger linewidth narrowing factor. Since Γ_b is usually larger than 10 MHz and γ is smaller than 1 MHz for our MMF microresonator, a linewidth narrowing factor of more than 100 (20 dB) is expected, as verified by our previous experimental results [12].

For SBL soliton, the fundamental comb linewidth is the same with the SBL [13]. Despite the compromised SBL linewidth narrowing factor by 3-5 times from the detuning effect [14], the SBL comb linewidth narrowing effect from the pump can still be larger than 20 dB.

SBL soliton jitter reduction The detuning is key to soliton's performance, including the soliton pulse width, soliton energy and so on. The detuning instability will cause the soliton jitter through fluctuation of Kerr nonlinearity from the affected soliton's pulse width and energy. Therefore the detuning noise suppression can lead to soliton jitter reduction.

According to the theoretical analysis [15], the relationship between the SBL detuning σ_B and pump detuning σ_F is described as

$$\sigma_B = \frac{\sigma_F - [(\Omega_b^2 - \Omega^2)/2\Omega]}{1 + \Gamma_b/\gamma} \tag{S5}$$

where Ω_b is the coustic frequency at SBS gain peak and Ω is the actual SBS frequency shift. Therefore, the SBL detuning noise can be largely suppressed from $d\sigma_B = d\sigma_F/(1 + \Gamma_b/\gamma)$. Besides, larger Q factor can lead to larger SBL detuning noise suppression thus the SBL soliton jitter reduction.

VIII. Limit of laser and comb frequency noise

In Fig. S3 we compare the fundamental linewidth and RIN of the single-frequency pump in the same microresonator-filtered laser nested by different microresonators with different Q factors of 2.6×10^8 and 2×10^7 and similar FSRs of ~10 GHz. The high-Q microresonator is made of graded index multimode fiber (GIF50E, Thorlabs) while the low-Q microresonator is made of highly nonlinear fiber. No SBS effect is found, and only single frequency laser can oscillate for the two Chimera cavities. In addition, both lasers are operated at low power level to exclude the Kerr effect.

Since the microresonator-filtered laser can be viewed as a self-injection locked laser [16] via the microresonator, in theory higher Q should lead to better laser frequency noise suppression [17]. In Fig. S3a, the frequency noises of the two single-frequency lasers are counterintuitively almost identical despite the different Q factors. Therefore, the laser frequency noise is believed to be limited by other factors. According to the measured laser RINs with almost the same frequency spectra in Fig. S3b, we believe the laser frequency noise is limited by the laser RIN which is eventually dominated by the EDFA pump.

In Fig. 5b in the main text, the self-injection locked pump laser shares almost the same RIN with the excited SBL. Since in the microresonator the RIN suppression and frequency noise suppression are correlated during the SBS process [15], the linewidth narrowing factor of the SBL compared to the pump is expected to be near unit, which is verified by the fundamental linewidths in Fig. 5a in the main text. In other words, the pump RIN limits the frequency noise of both the pump and the SBL due to the conversion from amplitude noise to phase noise. To improve the pump RIN, EDFA pumps with better RIN should be employed.

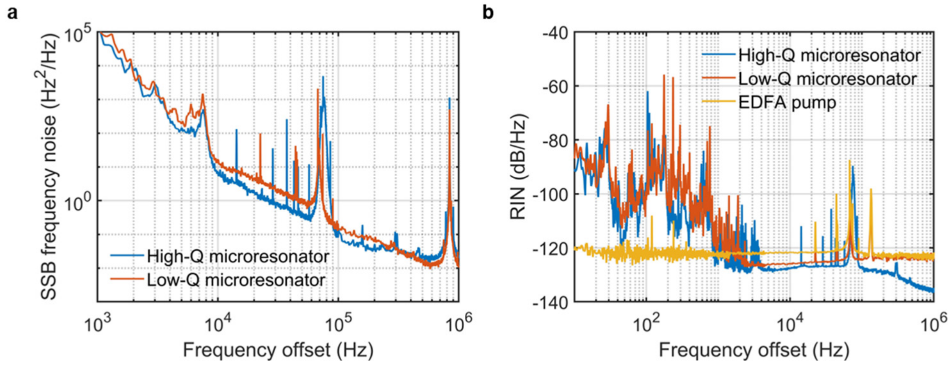

Fig. S11. Noise performance comparison for different microresonator-filtered lasers. (a) fundamental linewidth and (b) RIN of the single-frequency laser in microresonator-filtered lasers nested by microresonators with different Q factors.

IX. Analysis on the phase noise of the generated microwave signal

SBS frequency shift According to the frequency noise measurement in Fig. 5a and Fig. S3a, the laser phase noise PSDs of both pump and SBL follow $1/f^4$ trend with the offset frequencies and are dominated by the pump RIN as shown in Section VIII. Therefore, the phase noise PSD of the SBS frequency shift sees noise suppression of 10 dB/decade from the pump or SBL phase noise and follows $1/f^3$ trend with the offset frequencies as shown in Fig. 5c. The phase noise PSD with $1/f^3$ trend is mainly dominated by frequency flicker noise resulting from pump laser frequency drift. Despite the common mode noise suppression of 10 dB/decade, the coherence between pump and SBL is still worse compared to the one between phase locked SBL comb lines. Of note, for the external pumped case [12], the phase noise PSD of the SBS frequency shift is dominated by the pump laser phase noise and no noise suppression with respect to the pump laser is observed [12].

SBL soliton comb repetition rate As demonstrated in Section VII, the soliton detuning noise is largely suppressed due to the SBS process, leading to reduced timing jitter. We measure the SSB phase noise of the soliton repetition rate with an all-fiber reference-free Michelson interferometer (ARMI) timing jitter measurement apparatus. The error signal is fed back to the PZT stretcher in the fiber link, and the timing delay provided by the long fiber link is locked to the free-running comb. As a result, the timing jitter PSD of the free-running comb can be measured outside the locking bandwidth [18,19]. It is important to keep the locking bandwidth as low as possible to ensure broadband characterization. In our work, we used a 100 Hz locking bandwidth so that the SSB phase noise can be precisely measured from 100 Hz to higher offset frequencies.

As shown Fig. 5c, the phase noise PSD of the comb repetition rate follows $1/f^2$ trend with the offset frequencies, which means white frequency noise of the generated microwave signal. However, the phase noise PSD is still 15 dB larger than the calculated quantum noise limit (Fig. S12) [12,20]. According to our previous analysis [12], we attribute that the phase noise of the SBL soliton comb repetition rate is dominated by the SBL RIN and pump RIN, which depends on the 980-nm laser RIN as analyzed in Section II. In particular, for the offset frequencies below 1 kHz, the phase noise of the comb repetition rate is dominated by the technical noise from the EDFA whose gain response time is around ms level. Of note, the SSB phase noise below 10 kHz can be easily reduced by a feedback loop.

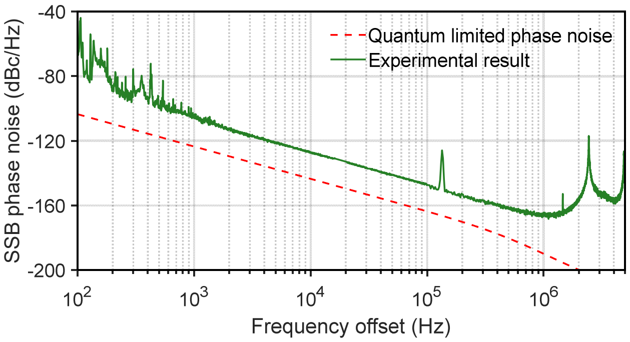

Fig. S12. Quantum noise limit for our MMF microresonator. The red dashed line shows the quantum limited phase noise of the comb, which is still much lower than the experimental result.

In terms of long-term stability, the key is the pump laser frequency which can be affected by the large fiber cavity length, the temperature of the microresonator, the gain stability in the EDFA and so on. One of the examples is in Fig. 4 we show the dynamics of comb power and repetition rate when we modulate the large fiber cavity length. Therefore, we can improve the long-term stability by shielding the whole system, using high-precision temperature controller, introducing feedback on the 980-nm laser and so on.

X. Noise performance comparison

Fundamental comb linewidth Table S1 lists the fundamental comb linewidth in different platforms and with different methods. According to Table 1, in general, our SBL soliton comb linewidth shows the best result among the reported results.

Table S1. Comparison of DKS microcomb linewidth

Material	Q factor (Million)	Microcomb linewidth (Hz)	Soliton active control	Reference
SiO_2	278	~0.1	W/o (microresonator-filtered laser)	This work

SiO_2	384	~0.4	W/o (SBS)	[12]
SiO_2	44.4	24	W/o (SBS)	[21]
Si_3N_4	56	~10	W/o (SIL*)	[17]
Si_3N_4	11.6	~1000	W/	[22]

*SIL: self-injection locking.

For SBL soliton, the fundamental comb linewidth is the same with the SBL [13]. Based on the analysis in Section VII, the SBL linewidth narrowing factor depends on both the Q factors and SBS gain bandwidth. Therefore, larger Q factors and larger SBS gain bandwidth can lead to lower SBL and its comb fundamental linewidth. For example, in this work with microresonator-filtered laser and our previous SBL soliton comb pumped by an ECDL [12], the MMF microresonator has larger Q factors of ~300 million, resulting in sub-hertz fundamental comb linewidth. However, for the silica disk microresonator [21], the Q factor is 44.4 million, which is 10 times lower than our MMF microresonator and this results in 24-Hz comb fundamental linewidth due to the smaller linewidth narrowing factor.

For the soliton achieved by the self-injection locking method, the comb fundamental linewidth is usually determined by the Q-factor [17] and is eventually limited by the small mode volume induced large thermo-refractive noise for the on-chip microresonators [17]. For example, for the on-chip Si_3N_4 microresonator with high Q factor of 56 million, the fundamental comb linewidth is ~10 Hz which is limited by the thermo-refractive noise.

The pump laser phase noise suppression in our microresonator-filtered laser can also be viewed as a result of self-injection locking, which is discussed in detail in Section VIII. The ultrahigh Q factor of ~300 million is responsible for the 0.1-Hz fundamental linewidth for the pump laser. Besides, the pump RIN eventually limits the pump laser fundamental linewidth to be lower than 0.1 Hz.

Comb repetition rate Table S2 lists the state-of-the-art phase noise of DKS microcomb repetition rate in various platforms with different methods. According to Table 2, in general, our SBL soliton jitter (either in microresonator-filtered laser or externally pumped) in MMF microresonator, outperforms all other DKS microcombs, with or without active control, except the one study in a MgF_2 crystalline microresonator [23] where a sideband Pound–Drever–Hall locking was implemented to optimize and stabilize the detuning setpoint via the ultrastable laser for the quiet point (QP) operation that requires either avoided mode crossing or large third order dispersion. Our approach based on SBS process in MMF microresonator, on the other hand, is completely free running without the need of any active control.

Table S2. Comparison of phase noise of DKS microcomb repetition rate

Material	Configuration	Carrier frequency (GHz)	SSB phase noise (dBc/Hz, scaled to 10 GHz)		Soliton active control	Reference
			10 kHz	100 kHz		
SiO_2	Bright soliton	10.08/20.16	-128	-147	W/o (microresonator -filtered laser)	This work
SiO_2	Bright soliton	10.08/20.16	-125	-148	W/o (SBS)	[12]
SiO_2	Bright soliton	0.945	-120	-140	W/o (SBS)	[11]
SiO_2	Bright soliton	10.43	-125	-144	W/o (SBS)	[21]
SiO_2	Bright soliton	11.02	-120	-139	W/o (SBS)	[24]
Si_3N_4	Dark soliton	5.4	-108	-134	W/o (SIL)	[17]
MgF_2	Bright soliton	9.9	-130	-130	W/o (SIL)	[25]
SiO_2	Bright soliton	22	-111	-147	W/ (QP)	[19]
SiO_2	Bright soliton	15.2	-117	-143	W/ (QP)	[26]
SiO_2	Bright soliton	11.4	-134	-143	W/ (QP)	[27]

MgF_2	Bright soliton	14.09	-142	-159	W/ (QP)	[23]
Si_3N_4	Bright soliton	9.78	-110	-130	W/	[28]

*SIL: self-injection locking.

Our previous SBL soliton jitter in a monolithic fiber resonator based on highly nonlinear fiber reaches the quantum noise limit [11]. Here the MMF microresonator in this work has two-fold advantages, the large mode volume and lower Kerr nonlinearity parameter, which can efficiently lower the quantum noise limit [20]. Therefore, the SBL soliton jitter in this work and our recent work [12] is not limited by the quantum noise, but the SBL RIN, as analyzed in Section IX. Besides, according to the detuning noise suppression analysis in Section VII, higher Q factor will lead to better detuning noise suppression thus lower soliton jitter. This should be the reason why our soliton jitter results with ultrahigh-Q MMF microresonator outperforms the other SBL soliton jitter results even though they are not limited by the quantum noise.

XI. Soliton long-term stability

We show in Fig. S13 the long-term stability of pump detuning and pump frequency shift during the 2-h free-running operation.

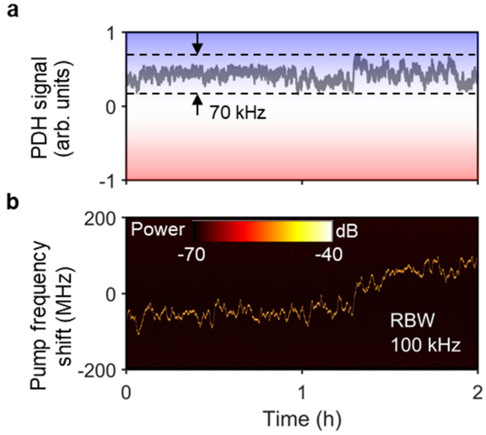

Fig. S13. Long-term stability of pump detuning and pump frequency shift. (a) Temporal evolution of pump PDH signal. (b) Temporal evolution of pump laser frequency.

XII. PDH error signal

We show in Fig. S14 how we experimentally obtained the PDH error signal in the microresonator-filtered laser (a) and in the externally pumped configuration (b).

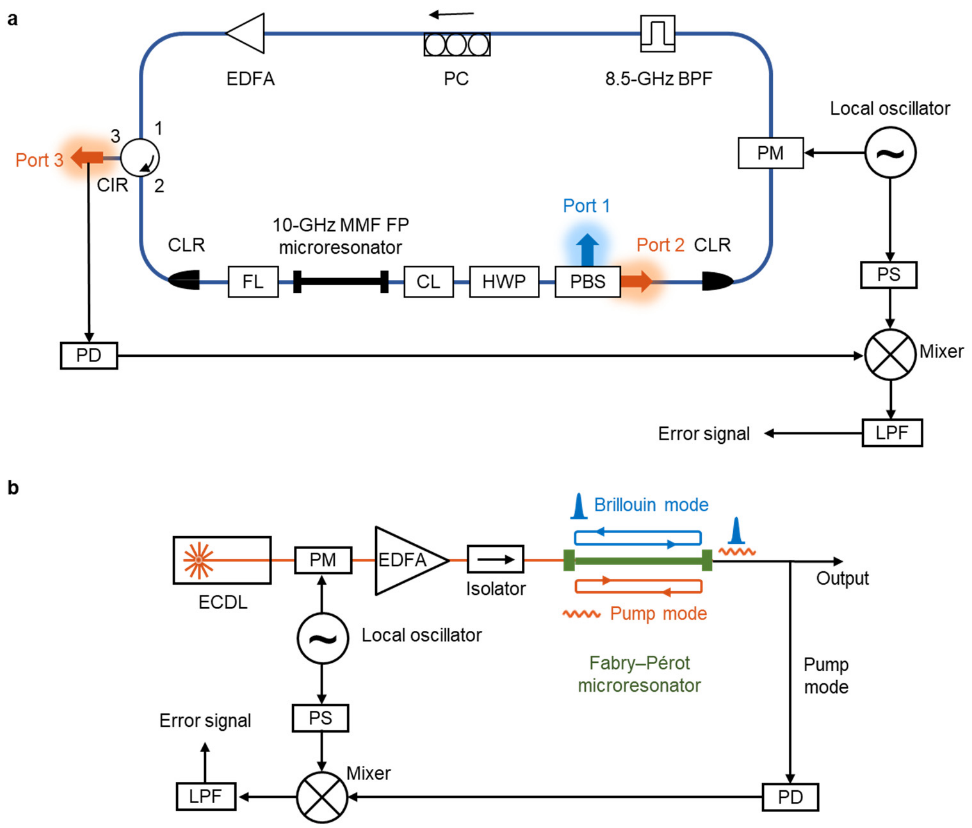

Fig. S14. Measurement of PDH error signal. (a) in the microresonator-filtered laser. (b) in the externally pumped configuration. PD: photodetector; PM: phase modulator; PS: phase shifter; LPF: low-pass filter.

Supplementary References

[1] T. Carmon, L. Yang, K.J. Vahala, Dynamical thermal behavior and thermal self-stability of microcavities, Opt. Express. 12 (2004) 4742–4750. https://doi.org/10.1364/OPEX.12.004742.

[2] W. Zou, Z. He, K. Hotate, Complete discrimination of strain and temperature using Brillouin frequency shift and birefringence in a polarization-maintaining fiber, Opt. Express. 17 (2009) 1248–1255. https://doi.org/10.1364/OE.17.001248.

[3] Y. Bao, G. Chen, Temperature-dependent strain and temperature sensitivities of fused silica single mode fiber sensors with pulse pre-pump Brillouin optical time domain analysis, Meas. Sci. Technol. 27 (2016) 065101. https://doi.org/10.1088/0957-0233/27/6/065101.

[4] H. Zhou, Y. Geng, W. Cui, S.-W. Huang, Q. Zhou, K. Qiu, C.W. Wong, Soliton bursts and deterministic dissipative Kerr soliton generation in auxiliary-assisted microcavities, Light Sci. Appl. 8 (2019) 1–10.

[5] Y. Zhao, L. Chen, C. Zhang, W. Wang, H. Hu, R. Wang, X. Wang, S.T. Chu, B. Little, W. Zhang, others, Soliton burst and bi-directional switching in the platform with positive thermal-refractive coefficient using an auxiliary laser, Laser Photonics Rev. 15 (2021) 2100264. https://doi.org/10.1002/lpor.202100264.

[6] S. Zhang, J.M. Silver, L. Del Bino, F. Copie, M.T. Woodley, G.N. Ghalanos, A.Ø. Svela, N. Moroney, P. Del'Haye, Sub-milliwatt-level microresonator solitons with extended access range using an auxiliary laser, Optica. 6 (2019) 206–212. https://doi.org/10.1364/OPTICA.6.000206.

[7] Y. Zheng, C. Sun, B. Xiong, L. Wang, Z. Hao, J. Wang, Y. Han, H. Li, J. Yu, Y. Luo, others, Soliton dynamics in microresonators with XPM induced negative thermo-optic effect, ArXiv Prepr. ArXiv201100687. (2020).

[8] Z. Lu, H.-J. Chen, W. Wang, L. Yao, Y. Wang, Y. Yu, B. Little, S. Chu, Q. Gong, W. Zhao, others, Synthesized soliton crystals, Nat. Commun. 12 (2021) 1–7. https://doi.org/10.1038/s41467-021-23172-2.

[9] I.S. Grudinin, A.B. Matsko, L. Maleki, Brillouin lasing with a CaF 2 whispering gallery mode resonator, Phys. Rev. Lett. 102 (2009) 043902. https://doi.org/10.1103/PhysRevLett.102.043902.

[10] D. Braje, L. Hollberg, S. Diddams, Brillouin-enhanced hyperparametric generation of an optical frequency comb in a monolithic highly nonlinear fiber cavity pumped by a cw laser, Phys. Rev. Lett. 102 (2009) 193902. https://doi.org/10.1103/PhysRevLett.102.193902.
[11] K. Jia, X. Wang, D. Kwon, J. Wang, E. Tsao, H. Liu, X. Ni, J. Guo, M. Yang, X. Jiang, others, Photonic flywheel in a monolithic fiber resonator, Phys. Rev. Lett. 125 (2020) 143902.
[12] M. Nie, K. Jia, Y. Xie, S. Zhu, Z. Xie, S.-W. Huang, Synthesized spatiotemporal mode-locking and photonic flywheel in multimode mesoresonators, Nat. Commun. 13 (2022) 1–9. https://doi.org/10.1038/s41467-022-34103-0.
[13] P. Liao, C. Bao, A. Kordts, M. Karpov, M.H. Pfeiffer, L. Zhang, A. Mohajerin-Ariaei, Y. Cao, A. Almaiman, M. Ziyadi, others, Dependence of a microresonator Kerr frequency comb on the pump linewidth, Opt. Lett. 42 (2017) 779–782. https://doi.org/10.1364/OL.42.000779.
[14] Z. Yuan, H. Wang, L. Wu, M. Gao, K. Vahala, Linewidth enhancement factor in a microcavity Brillouin laser, Optica. 7 (2020) 1150–1153.
[15] W. Loh, S.B. Papp, S.A. Diddams, Noise and dynamics of stimulated-Brillouin-scattering microresonator lasers, Phys. Rev. A. 91 (2015) 053843. https://doi.org/10.1103/PhysRevA.91.053843.
[16] A. Pasquazi, M. Peccianti, L. Razzari, D.J. Moss, S. Coen, M. Erkintalo, Y.K. Chembo, T. Hansson, S. Wabnitz, P. Del'Haye, others, Micro-combs: A novel generation of optical sources, Phys. Rep. 729 (2018) 1–81. https://doi.org/10.1016/j.physrep.2017.08.004.
[17] W. Jin, Q.-F. Yang, L. Chang, B. Shen, H. Wang, M.A. Leal, L. Wu, M. Gao, A. Feshali, M. Paniccia, others, Hertz-linewidth semiconductor lasers using CMOS-ready ultra-high-Q microresonators, Nat. Photonics. 15 (2021) 346–353.
[18] D. Kwon, C.-G. Jeon, J. Shin, M.-S. Heo, S.E. Park, Y. Song, J. Kim, Reference-free, high-resolution measurement method of timing jitter spectra of optical frequency combs, Sci. Rep. 7 (2017) 1–9.
[19] D. Jeong, D. Kwon, I. Jeon, I.H. Do, J. Kim, H. Lee, Ultralow jitter silica microcomb, Optica. 7 (2020) 1108–1111.
[20] A.B. Matsko, L. Maleki, On timing jitter of mode locked Kerr frequency combs, Opt. Express. 21 (2013) 28862–28876.
[21] Y. Bai, M. Zhang, Q. Shi, S. Ding, Y. Qin, Z. Xie, X. Jiang, M. Xiao, Brillouin-Kerr soliton frequency combs in an optical microresonator, Phys. Rev. Lett. 126 (2021) 063901.
[22] F. Lei, Z. Ye, Ó.B. Helgason, A. Fülöp, M. Girardi, V. Torres-Company, Optical linewidth of soliton microcombs, Nat. Commun. 13 (2022) 1–9. https://doi.org/10.1038/s41467-022-30726-5.
[23] E. Lucas, P. Brochard, R. Bouchand, S. Schilt, T. Südmeyer, T.J. Kippenberg, Ultralow-noise photonic microwave synthesis using a soliton microcomb-based transfer oscillator, Nat. Commun. 11 (2020) 1–8. https://doi.org/10.1038/s41467-019-14059-4.
[24] I.H. Do, D. Kim, D. Jeong, D. Suk, D. Kwon, J. Kim, J.H. Lee, H. Lee, Self-stabilized soliton generation in a microresonator through mode-pulled Brillouin lasing, Opt. Lett. 46 (2021) 1772–1775.
[25] W. Liang, D. Eliyahu, V.S. Ilchenko, A.A. Savchenkov, A.B. Matsko, D. Seidel, L. Maleki, High spectral purity Kerr frequency comb radio frequency photonic oscillator, Nat. Commun. 6 (2015) 1–8. https://doi.org/10.1038/ncomms8957.
[26] Q.-F. Yang, Q.-X. Ji, L. Wu, B. Shen, H. Wang, C. Bao, Z. Yuan, K. Vahala, Dispersive-wave induced noise limits in miniature soliton microwave sources, Nat. Commun. 12 (2021) 1–10.
[27] L. Yao, P. Liu, H.-J. Chen, Q. Gong, Q.-F. Yang, Y.-F. Xiao, Soliton microwave oscillators using oversized billion Q optical microresonators, Optica. 9 (2022) 561–564. https://doi.org/10.1364/OPTICA.459130.
[28] J. Liu, E. Lucas, A.S. Raja, J. He, J. Riemensberger, R.N. Wang, M. Karpov, H. Guo, R. Bouchand, T.J. Kippenberg, Photonic microwave generation in the X- and K-band using integrated soliton microcombs, Nat. Photonics. 14 (2020) 486–491. https://doi.org/10.1038/s41566-020-0617-x.
