# Supplementary material for: Turnkey photonic flywheel in a microresonator-filtered laser
Source: Nat Commun. 2024 Jan 2;15:55. doi: 10.1038/s41467-023-44314-8 (PMC10761980; doi:10.1038/s41467-023-44314-8)
Supplement: Supplementary file 3 — Description of Additional Supplementary Files [file 41467_2023_44314_MOESM3_ESM.pdf]

<u>Description of Additional Supplementary Files</u>

Supplementary Movie S1: Numerical demonstration of self-starting soliton in a microresonator-filtered fiber laser

Supplementary Movie S2: Cavity attractor under different SBS frequency shifts
