# Supplementary material for: Turnkey photonic flywheel in a microresonator-filtered laser
Source: Nat Commun. 2024 Jan 2;15:55. doi: 10.1038/s41467-023-44314-8 (PMC10761980; doi:10.1038/s41467-023-44314-8)
Supplement: Supplementary file 6 — Reporting Summary [file 41467_2023_44314_MOESM6_ESM.pdf]

Corresponding author(s): Shu-Wei Huang

Lasing Reporting Summary

Nature Research wishes to improve the reproducibility of the work that we publish. This form is intended for publication with all accepted papers reporting claims of lasing and provides structure for consistency and transparency in reporting. Some list items might not apply to an individual manuscript, but all fields must be completed for clarity.

For further information on Nature Research policies, including our data availability policy, see Authors & Referees.

Experimental design

Please check: are the following details reported in the manuscript?

1. Threshold

Plots of device output power versus pump power over a wide range of values indicating a clear threshold

☒ Yes ☐ No

Subsection "Details of experimental setup" in the Methods section.

2. Linewidth narrowing

Plots of spectral power density for the emission at pump powers below, around, and above the lasing threshold, indicating a clear linewidth narrowing at threshold

☐ Yes ☒ No

Lasing in a microresonator-filtered laser is well known. Instead, we showed the linewidth narrowing of the stimulated Brillouin lasing and Brillouin-dissipative Kerr soliton comb (Fig. 5), in a good agreement with the theory.

Resolution of the spectrometer used to make spectral measurements

☒ Yes ☐ No

Subsection "Measurement of laser phase noise and fundamental linewidth" in the Methods section.

3. Coherent emission

Measurements of the coherence and/or polarization of the emission

☒ Yes ☐ No

Figs. 1, 3, and 5

4. Beam spatial profile

Image and/or measurement of the spatial shape and profile of the emission, showing a well-defined beam above threshold

☒ Yes ☐ No

Figure 1

5. Operating conditions

Description of the laser and pumping conditions

Continuous-wave, pulsed, temperature of operation

☒ Yes ☐ No

Subsection "Details of experimental setup" in the Methods section.

Threshold values provided as density values (e.g. W cm^{-2} or J cm^{-2}) taking into account the area of the device

☒ Yes ☐ No

Subsection "Details of experimental setup" in the Methods section.

6. Alternative explanations

Reasoning as to why alternative explanations have been ruled out as responsible for the emission characteristics

e.g. amplified spontaneous, directional scattering; modification of fluorescence spectrum by the cavity

☒ Yes ☐ No

The low-noise comb spectra characterized in Figs. 3 and 5 rule out the alternative explanations.

7. Theoretical analysis

Theoretical analysis that ensures that the experimental values measured are realistic and reasonable

e.g. laser threshold, linewidth, cavity gain-loss, efficiency

☒ Yes ☐ No

Subsection "Principle of turnkey Brillouin-DKS" and Supplemental Information Section III.

8. Statistics

Number of devices fabricated and tested

☐ Yes ☒ No

This is a proof-of-principle demonstration and thus the study focus is the underlying physics rather than the performance statistics.

Statistical analysis of the device performance and lifetime (time to failure)

☐ Yes ☒ No

This is a proof-of-principle demonstration and thus the study focus is the underlying physics rather than the performance statistics.
